# Engaging people with lived experience of dementia in research meetings and events: insights from multiple perspectives

**DOI:** 10.3389/frdem.2024.1421737

**Published:** 2024-07-05

**Authors:** Ellen Snowball, Christine Aiken, Myrna Norman, Wayne Hykaway, Zoe Dempster, Inbal Itzhak, Emily McLellan, Katherine S. McGilton, Jennifer Bethell

**Affiliations:** ^1^Knowledge, Innovation, Talent, Everywhere (KITE) Research Institute, Toronto Rehabilitation Institute, University Health Network, Toronto, ON, Canada; ^2^Engagement of People with Lived Experience of Dementia Program/Advisory Group, Canadian Consortium on Neurodegeneration in Aging, Montreal, QC, Canada; ^3^Knowledge Translation and Exchange Program, Canadian Consortium on Neurodegeneration in Aging, Montreal, QC, Canada; ^4^Lawrence S. Bloomberg Faculty of Nursing, University of Toronto, Toronto, ON, Canada; ^5^Institute of Health Policy, Management & Evaluation, Dalla Lana School of Public Health, University of Toronto, Toronto, ON, Canada

**Keywords:** dementia, aging, patient and public engagement, lived experience of dementia, health research, engagement in research, multi-stakeholder, advisory group

## Abstract

This perspective article describes the experiences of engaging people with lived experience of dementia in research meetings and events from the perspectives of people with lived experience, researchers, trainees, audience members and others. We outline examples of engagement from different events and describe a video project, initiated by people with lived experience, conveying diverse views about becoming integral collaborators in the Canadian Consortium on Neurodegeneration in Aging (CCNA) annual Partners Forum and Science Days. We also report evaluation data from audiences and present a series of tips and strategies for facilitating this engagement, including practical considerations for supporting people with lived experience.

## 1 Introduction

Dementia describes the symptoms related to neurodegenerative conditions, such as Alzheimer's disease, vascular dementia, Lewy body dementia, and others. These symptoms include memory loss, difficulties in thinking, problem-solving and language, and changes in mood and behavior. Dementia can impact a person's ability to perform everyday activities, such as bathing, dressing and cooking (Cipriani et al., 2020). Risk increases with age and most of those living with dementia are older adults (Canadian Institute for Health Information, 2015). Dementia is highly stigmatized (Link and Phelan, 2001). Stigmas associated with dementia, compounded by impacts of ageism and ableism, threaten social participation of people living with dementia as well as their family and friends and can be a barrier to care and support (Vernooij-Dassen et al., 2005; Werner et al., 2014; Herrmann et al., 2018).

Increasingly, patient engagement^1^ in research is required by funding agencies, including in the United States, Canada, the United Kingdom (Forsythe et al., 2015; Manafo et al., 2018). The concept, rooted in HIV/AIDS research and the disability rights movement, asserts that individuals affected by publicly funded research have the right to actively participate in it (Shimmin et al., 2017). It has also been suggested to lead to better quality research with greater impact (Domecq et al., 2014; Wilson et al., 2015; Chudyk et al., 2022; Marshall et al., 2023). In the context of patient engagement in research, people with lived experience are taking on roles such as co-applicants on grants, research team members, co-authors on papers and others (Bethell et al., 2018; Snowball et al., 2022).

While much has been written about the motivations for and benefits of patient engagement, less is known about the potential challenges and risks to people with lived experience. Patient engagement activities that are not conducted ethically can pose distinct risks to people with lived experience, such as experiences of tokenism, stigmatization, re-traumatization, power imbalance, and discrimination (Hahn et al., 2016; Government of Canada, 2020; Richards et al., 2023; Zubair, 2023). Moreover, similar to participation in research on dementia (Vyas et al., 2018), racialized individuals and other marginalized groups are under-represented in patient engagement activities (Keane et al., 2023), thereby perpetuating experiences of discrimination. These experiences can harm the individual, and/or leave them disillusioned with research (Richards et al., 2023). Recommendations for patient engagement approaches, such as using anti-oppressive frameworks, would help facilitate meaningful engagement that supports the dignity and personhood of people with lived experience (Kontos, 2005; Cowdell, 2006; Kontos et al., 2017; Ontario's Patient Engagement Framework, 2017; Shimmin et al., 2017; Government of Canada, 2018, 2020; Roche et al., 2020; University Health Network, 2023; Zubair, 2023). However, there remain gaps in the literature on best practices, from the point of view of people with lived experience and specific to different research roles, venues (Poitras et al., 2020) and populations being engaged.

This article aims to describe experiences of engagement from the perspectives of people with lived experience of dementia, researchers and others, on collaborating in research meetings and events. We outline examples of engagement from different events and activities, including a video project, initiated by people with lived experience, conveying diverse views about becoming integral collaborators in the Canadian Consortium on Neurodegeneration in Aging (CCNA) annual conference. We also report evaluation data from audiences and present a series of tips and strategies for facilitating engagement in these contexts, including practical considerations for supporting people with lived experience in research events and meetings. These descriptions and findings, however, are limited to the experiences of those living with early stage dementia together with friends, family and care partners/caregivers who have collectively experienced early, middle and late stage dementia. We hope this paper will support people with lived experience in research and those seeking to involve them in similar settings.

### 1.1 Engagement of People with Lived Experience of Dementia Advisory Group and Cross–cutting Program

CCNA was developed to advance research on neurodegenerative diseases. It is a pan-Canadian network funded by the Canadian Institutes of Health Research and partner organizations. CCNA researchers are supported by cross-cutting programs, including the Engagement of People with Lived Experience of Dementia (EPLED)—introduced in CCNA Phase II (starting in 2019).

EPLED's objectives are to: (1) Support persons with dementia and care partners to be involved in the research process; (2) Work with research teams, cross-cutting programs and partners to develop novel mechanisms to further this collaboration; and to (3) Advance the methods of patient engagement in research through evaluation. EPLED is co-led by two academic researchers (JB and KMcG), managed by a research associate (ES), and funded by the Alzheimer Society of Canada.

In 2020, EPLED developed an Advisory Group of individuals, from across Canada, with diverse lived experiences of dementia (e.g., people living with dementia, friends, family and care partners/caregivers) who would work with CCNA researchers—not as study subjects but as collaborators in research (Snowball et al., 2022). EPLED has worked to integrate the lived experience Advisory Group members in various initiatives and to meaningfully and actively involve them in research activities.

## 2 Activities and roles

### 2.1 Canadian Consortium on Neurodegeneration in Aging Partners Forum and Science Days

CCNA Partners Forum and Science Days (PFSD) are venues to share research within the network. Previously held annually and in-person, the conference moved online due to COVID-19. In 2020, the conference agenda included a workshop to introduce EPLED. In 2021, to increase integration, EPLED Advisory Group members were invited to the planning committee. Members provided feedback on session ideas and developed roles within the program. The resulting conference agenda included two panels featuring three Advisory Group members; one about collaborating on an international research project and another about social connection and long-term care homes. In 2022, we deliberately shifted away from a lived-experience-focused session as attendance was primarily researchers already committed to patient engagement. Instead, we worked to integrate lived experience perspectives across the scientific program, including by creating new roles for members that prioritized their voices. For example, a person with dementia spoke on an opening session panel alongside CCNA's Scientific Director and Canada's Minister of Health, and a caregiver delivered the closing session. In the regular sessions, Advisory Group members participated as speakers alongside researchers and in a discussant role, where they could pose the first questions from the audience. There were other opportunities to share lived experience stories through a series of recorded videos.

### 2.2 Canadian Consortium on Neurodegeneration in Aging Public Events

CCNA Public Events are venues for sharing research with non-scientific audiences. In 2020, these events moved online due to COVID-19. Advisory Group members joined the planning committee in 2021. They discussed addressing the needs of care partners/caregivers, and so the event focused on “Caring and Caregiving for a Person Living with Dementia”. An EPLED Advisory Group member participated as a panelist speaker alongside three researchers. EPLED and CCNA staff worked with them to prepare a recorded message for attendees. In 2022, recognizing EPLED's impact, five Advisory Group members joined the planning committee. They created a focus for the event, “Finding Hope in Dementia”, around practical ways to live well with dementia. The panel included two researchers and two Advisory Group members. The webinar was structured using informal conversation and members spoke about quality of life and strategies for finding hope.

### 2.3 Canadian Institutes of Health Research—Institute of Aging Summer Program in Aging

In 2022, an EPLED co-lead (JB) joined the program planning committee at the Canadian Institutes of Health Research—Institute of Aging Summer Program in Aging (SPA). Advisory Group members participated in the conference program; eight joined 30-min “Coffee Breaks” with trainees, and three spoke in program sessions. An open format was used, where trainees could ask questions about EPLED engagement. These sessions were short, allowing trainees to join in-between other sessions.

### 2.4 Vascular training platform conference

In 2023, The Vascular Training (VAST) program integrated lived experience into their first annual in-person conference. Three EPLED Advisory Group members and one EPLED staff member (ES) were invited to join the planning committee. Advisory Group members envisioned a panel on how researchers can engage people with lived experience throughout the research process. They invited a biomedical researcher who had prior experience collaborating with them to speak from a researcher perspective. The panel was presented to an in-person research audience in Montreal, Quebec. It featured four Advisory Group members; two caregivers and two people living with dementia. Members spoke about their experiences collaborating in research, including impact on research, and barriers and enablers to engagement.

## 3 Methods

### 3.1 Evaluation data

Evaluation data were collected in online, anonymous surveys using a 5-point Likert scale (rating the helpfulness or meaningfulness of lived experience perspectives or enhanced awareness of benefits of lived experience involvement) and/or via open-ended questions (Table 1).

**Table 1:** Event evaluation data collected after Advisory Group collaborations.

**Event/audience**	**Evaluation question**	**Mean score**	**Feedback**
CCNA PFSD conference (2022)/Primarily researchers, including trainees (*n* = 80 responses)	“This session featured a member from CCNA's Engagement of People with Lived Experience of Dementia (EPLED) Advisory Group. How helpful was it to hear their perspective?”	•Opening (Session 1): 4.8 out of 5 •Stress & Dementia (Session 5): 4.6 out of 5 •Closing (Session 18): 4.8 out of 5	*It was powerful to hear from someone affected by dementia who has worked in the field and is now passionate about patient engagement in research* (Session 1). *It was very helpful and moving to hear from someone with lived experience. It made the issue more real and not just an academic exercise* (Session 2). *Extremely helpful as it reminds us researchers of the importance not only to do research, publish studies and present them to conferences, but also to share the knowledge to the general public, to engage more with local groups and colleagues from other fields so that those living with dementia (and their caregivers) are never left alone and are offered all the help they deserve* (Session 18).
SPA conference (2022)/Trainees (*n* = 16 responses)	“SPA 2022 increased my awareness of the benefits of involving those with lived experience in research on age-related conditions associated with impaired cognition”	•4.6 out of 5 •Tied for highest rating with 9/16 saying they strongly agreed	*The most important and meaningful takeaways were the many lessons and discussions with people with lived experience. The primary motivation to do research on neurodegeneration is to help real people with real problems, not just articles for our own career's sake*.
CCNA Public Event (2022)/General public (*n* = 58 responses)	“One of the panel members, Linda, was a caregiver who shared her experience caring for her husband with dementia. Was it helpful to include a caregiver on the panel? Please explain.”	•N/A	*This was the most useful part of the presentation. Her lived experience made me feel less alone. She had excellent suggestions for advocacy and for caregiving. We can learn more from personal experience than a textbook. Oh my goodness - I learned the most from her!*
CCNA Public Event (2023)/General public (*n* = 54 responses)	“This webinar featured speakers with lived experience of dementia (a care-partner and a person with dementia). How helpful was it to hear their perspective?”	•4.7 out of 5	*It's the first time I heard a person with dementia speak about it from their perspective. Hearing first hand from a patient with dementia, speaking so eloquently and clearly, broke down all my prejudices and fears about dementia. “Lived experience” is the strongest way to express truth, to share truth, and to live truth*.
VAST conference (2023)/Researchers and trainees (*n* = 17 responses)	“The involvement of people with lived experience was meaningful to me” “What were your favorite and least favorite sessions?”	•99 out of 100 •10/17 respondents mentioned the EPLED panel specifically as their favorite	*We can't forget the real people our research will benefit, not just in the future, but now! Working with PWLE advances not just clinical practice, but also scientific discovery. [I] [gained] [a] better understanding of how to explain my work to people outside of academia*.

### 3.2 Experiences of EPLED Advisory Group members

#### 3.2.1 Tips and strategies for engaging people with lived experience in research meetings and events

EPLED Advisory Group members discussed their experiences collaborating in these research events. They compiled a series of tips and strategies to encourage and assist others who might be planning research meetings and events involving people with lived experience.

#### 3.2.2 “Successful integration of lived experience perspectives in national dementia research meetings”—Video project

*Unless you are in a situation, you cannot relate to it. You can think about what may have happened. You can try to relate, but unless you're there living it day-to-day, you don't see what's going on* –

Wayne Hykaway (1952–2024)

EPLED Advisory Group members prioritized sharing their experiences through a video project that would be accessible to diverse audiences (i.e., researchers, research funding organizations and the public, including people with lived experience). By choosing a video, they felt that more audiences would learn about the value of lived experience perspectives and strategies for supporting collaborations.

The video (https://vimeo.com/900182095) described how the EPLED Advisory Group became an important part of the CCNA community. CCNA and EPLED staff worked with Advisory Group members to develop a script and interview guide. Using open-ended questions, staff interviewed researchers, trainees, and EPLED Advisory Group members on their reflections and experiences collaborating in the CCNA conference. The recorded discussions were used to illustrate insights for researchers, research funding organizations and the public, including people with lived experience. The video shows how people with lived experience can take on multiple roles in research, and perceived benefits from the perspectives of people with lived experience, researchers and event attendees.

## 4 Results

### 4.1 Evaluation data

Evaluation data shows that collaborations in these venues were highly rated by different audiences for increased awareness of the value of lived experience perspectives in research, and meaningfulness and helpfulness of lived experience participation (Table 1).

### 4.2 Tips and strategies for engaging people with lived experience in research meetings and events

#### 4.2.1 Engage early and hold frequent meetings

Engaging EPLED Advisory Group members early in event planning meetings provided them with time to build relationships and trust with others and be meaningfully included in the planning process (Richards et al., 2023). It was important to consult with Advisory Group members on meeting time, frequency and length. Regular, online, bi-weekly or monthly one hour meetings helped to ensure that meeting agendas were not rushed, and that there was time to build rapport through informal conversation (Litherland et al., 2018; Vellani et al., 2023). Meetings were planned around the availability of EPLED Advisory Group members, accommodating for day jobs, caregiving responsibilities, and other needs and limitations (Burton et al., 2019).

#### 4.2.2 Provide support

Logistical support included providing email reminders of upcoming meetings, notes/recordings from meetings and assistance with forms (e.g., travel reimbursement). It also included technical support such as connecting to online meetings, troubleshooting computer problems and accessing documents (Novek and Wilkinson, 2017; Burton et al., 2019; Frank et al., 2020). Varied degrees of support were required in preparing for EPLED Advisory Group participation in meetings (e.g., preparing scripts or presentation materials). For in-person meetings, members sometimes required assistance with travel planning in advance, during and after events and, for some, a support person (e.g., friend or relative) traveled with them (Guidelines on Inclusive Travel Meetings for People with Dementia, 2024). During travel, EPLED provided a staff contact number for questions outside of business hours and collected emergency contact information. There was frequent contact between staff and Advisory Group members and opportunities to request one-on-one meetings if needed. Emotional support was provided through building relationships and trust with the EPLED and CCNA team as well as among the Advisory Group members. EPLED and the Advisory Group worked to recognize the vulnerability in sharing personal lived experiences by holding space for difficult discussions, validating people's feelings and focusing on individual strengths (Burton et al., 2019). The EPLED staff member (ES), dedicated to supporting the Advisory Group, has lived experience of dementia and Advisory Group members also brought relevant expertise to the group dynamics.

#### 4.2.3 Create multiple roles

EPLED remained flexible on the level and nature of Advisory Group involvement. Members collaboratively created roles tailored to their varied interests, priorities, preferences, motivations, and needs (Frank et al., 2020). Roles were diversified to increase participation for Advisory Group members and engage audiences. For example, discussant roles were introduced at conference sessions, where Advisory Group members were prepared to ask the first audience question. “EPLED stories” were also introduced, where EPLED Advisory Group members recorded a short message about their lived experience. Clear descriptions and orientation on expectations and responsibilities for roles was essential.

#### 4.2.4 Include diverse perspectives

EPLED Advisory Group members highlighted the importance of representing diverse experiences of dementia and caregiving, including with respect to age, ethnicity and gender identity. We used a consensus-based approach to reach agreement on roles, but prioritized the voices of those living with dementia. The EPLED Advisory Group collectively created a safe, trauma-informed, space to develop equitable partnerships, emphasizing trust, empathy, self-awareness, and relationship-building (Shimmin et al., 2017; Roche et al., 2020)^2^. We utilized an anti-oppressive, social justice and health equity lens to our work, recognizing vulnerability (e.g., in sharing personal lived experiences), promoting reflexivity (e.g., understanding unconscious bias), and embodied selfhood (e.g., agency beyond cognition) (Kontos, 2005; Kontos et al., 2017; Shimmin et al., 2017; Roche et al., 2020; Zubair, 2023). This approach extended to interactions in research meetings and events, where Advisory Group members recognized the vulnerability in sharing lived experiences and, even in instances of diverging opinions, supported one another in doing so. We practiced and encouraged active listening, welcoming critical feedback as opportunities for reflection and improvement.

#### 4.2.5 Plan for informal and formal interactions

Relational strategies, such as bi-directional communication (e.g., conversations), were valued by EPLED Advisory Group members (Metz et al., 2022). During both virtual and in-person events, they enjoyed opportunities to interact with researchers, trainees and fellow lived experience members. This was seen as a way to expand their networks and learn from others' perspectives. Informal conversation was welcomed during meetings and was integrated in the programs through scheduled social time (Novek and Wilkinson, 2017).

#### 4.2.6 Plan for frequent breaks

At online and in-person events, we planned for frequent breaks that were scheduled in agendas. For in-person meetings, we arranged private break spaces nearby, such as a quiet meeting room (Guidelines on Inclusive Travel Meetings for People with Dementia, 2024). EPLED Advisory Group members appreciated when events were held in hotels, as it allowed them to go back to their rooms as needed. We ensured that missed information was communicated as needed.

#### 4.2.7 Encourage participation

The tips and strategies described herein are intended to encourage participation of people with lived experience. In all capacities, it was important to empower EPLED Advisory Group members with the knowledge that their lived experience was expertise and that their input was valuable. In our experience, involvement by EPLED Advisory Group members also encouraged participation from all audiences by demonstrating that different perspectives were valued. EPLED Advisory Group members contributed to various sessions, although it was key to acknowledge that some were highly technical (Burton et al., 2019). Presenting to academic and non-academic audiences can be challenging but sessions involving people with lived experience helped researchers and trainees to develop this skill set (Biglieri, 2021; Richards et al., 2023).

#### 4.2.8 Provide compensation and prepay expenses

Offering compensation helps to recognize the expertise, time and contributions of people with lived experience (Litherland et al., 2018). Referring to patient engagement compensation guidelines can provide guidance, such as payment based on type of engagement (Government of Canada, 2019, 2022)^3^. However, compensation should also be individualized according to unique needs and circumstances. Further, payment for travel expenses should be reimbursed. To minimize out-of-pocket expenses incurred by EPLED Advisory Group members and wait time for reimbursement, we prepaid expenses to the extent possible by booking travel, arranging hotel rooms and ground transportation (Guidelines on Inclusive Travel Meetings for People with Dementia, 2024).

#### 4.2.9 Use accessible language and spaces

Using accessible, person-centered language in all communications and venues helped EPLED Advisory Group members to feel included. The EPLED Advisory Group provided recommendations that advised researchers and trainees to tailor their communications, including using language that was jargon and acronym-free and, where possible, circulating material within the group at least 1-week in advance of meetings (https://www.epled.ca/s/Suggestions-For-Researchers). For in-person events, dementia-friendly guidelines were helpful, such as choosing locations that were accessible (e.g., had ramps and elevators), had break spaces (e.g., close to hotel rooms or designated quiet rooms), and were familiar and close to parking and public transit (Parkes et al., 2022)^4^. Because people with dementia can experience sensory overstimulation, choosing venues with lower noise (e.g., carpeted floors), with evenly and well-lit spaces and using clear, large signage was helpful (Dewing, 2009). For online events, we used videoconference applications that had accessibility features, such as closed captioning and recording capabilities. We provided visual supports when needed and clear cues when moving onto one agenda item to the next.

#### 4.2.10 Evaluate from different perspectives

After meetings and events, organizers evaluated the contributions of the lived experience perspectives by inviting audience feedback. Typically, this consisted of brief online surveys that included questions about the perceived usefulness and impact of including people with lived experience in the program. We shared these data with Advisory Group members to recognize their contributions, expertise and growth as well as discuss opportunities for improvement.

### 4.3 Dissemination

**Figure 1:**
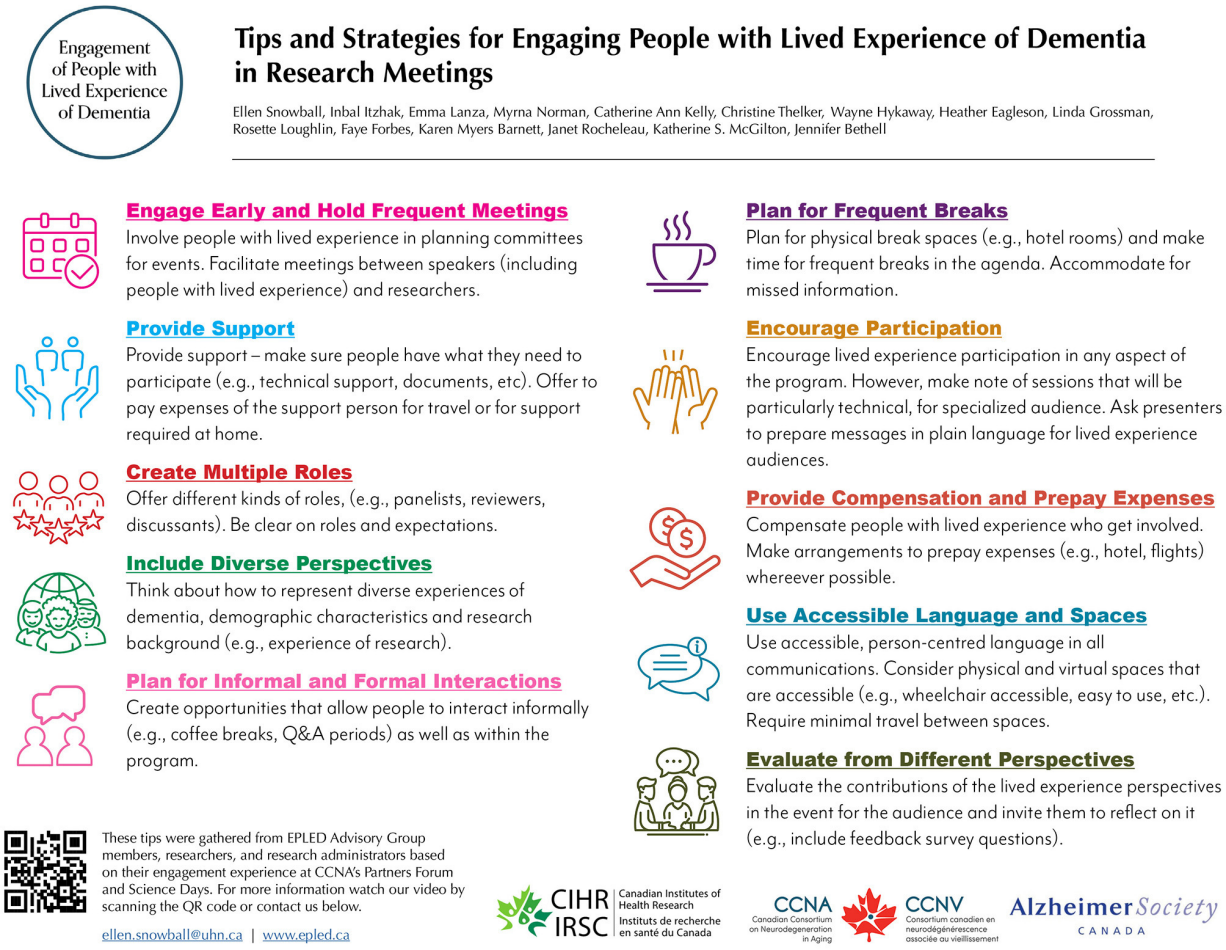
EPLED tip sheet infographic.

We posted the co-produced EPLED video and tip sheet infographic (Figure 1) online. We screened two versions of the video at the Pride in Patient Engagement in Research (PiPER) Research Day (October 2023 in Toronto): a 5-min version during a conference session, and the full 15-min video in a gallery space. We presented the infographic and evaluation data in a poster at the Canadian Association on Gerontology conference (October 2023 in Toronto). We also screened the 15-min version of the video and infographic at the Canadian Conference on Dementia (November 2023 in Toronto). In January 2024, EPLED and CCNA hosted a webinar, “‘Yes, It's Possible!': Top Tips for Engaging People with Lived Experience” (https://vimeo.com/905754604), featuring EPLED Advisory Group members, co-leads and CCNA staff. The video was screened during the opening session at CCNA Partners Forum and Science Days (March 2024 in Montreal).

## 5 Conclusion and future directions

In this perspective article, we described experiences of engaging people with lived experience of dementia in national research meetings and events. The article was written with people with lived experience who participated in those events, however, while this included people living with early stage dementia, we also acknowledge that perspectives of middle and late stage dementia were those of friends, family and care partners/caregivers. As patient engagement becomes more prominent in research, we anticipate an increase in resources on best practices on engaging diverse individuals and groups of people with lived experience, including those at different stages of dementia, racialized individuals and groups and 2SLGBTQIA+ communities. It is important that efforts in this area are informed by the perspectives of both researchers and people with lived experience. Guidelines that are not developed collaboratively, alongside people with lived experience, risk prioritizing academic perspectives and perpetuating negative experiences of tokenism and stigma. We hope this article can serve as a guide to those planning to engage people with lived experience in national research meetings and events.

## Data availability statement

The data analyzed in this study is subject to the following licenses/restrictions: the data were collected as part of program evaluation activities. Further inquiries can be directed to the corresponding author.

## Ethics statement

Ethical review and approval was not required for the study on human participants in accordance with the local legislation and institutional requirements. Written informed consent was not required to participate in this study in accordance with the local legislation and institutional requirements.

## Author contributions

ES: Conceptualization, Investigation, Project administration, Supervision, Visualization, Writing – original draft, Writing – review & editing. CA: Conceptualization, Writing – review & editing. MN: Conceptualization, Writing – review & editing. WH: Conceptualization, Writing – review & editing. ZD: Conceptualization, Writing – review & editing. II: Conceptualization, Writing – review & editing. EM: Conceptualization, Writing – review & editing. KM: Investigation, Writing – review & editing. JB: Conceptualization, Data curation, Funding acquisition, Investigation, Methodology, Project administration, Resources, Supervision, Visualization, Writing – original draft, Writing – review & editing.

## Footnotes

^1^Canadian Institute of Health Research (2019) defines patient engagement as: “an approach that involves meaningful and active collaboration in governance, priority setting, conducting research and knowledge translation” “Patient” is a term that refers to people with lived experience of a health issue. The authors acknowledge that using this term fails to account for people's full identities and experiences. However, for continuity, we refer to lived experience engagement in research as patient engagement throughout this paper.

^2^Diversity in Patient Engagement Learning Exchange. (2019). Available online at: https://www.healthcareexcellence.ca/media/xamlyars/dle-report-e-final-ua.pdf.

^3^Patient and Public Partner Appreciation Policy and Protocol, SPOR Evidence Alliance. (2022). Available online at: https://sporevidencealliance.ca/wp-content/uploads/2022/01/SPOREA_Patient-and-Public-Appreciation-Policy_2021.01.14-1.pdf.

^4^DEEP Guide Choosing a dementia-friendly meeting space (2013). Available online at: https://www.dementiavoices.org.uk/wp-content/uploads/2013/11/DEEP-Guide-Choosing-a-meeting-space.pdf.

## In memoriam

This paper is dedicated to the memory of Wayne Hykaway who passed away on May 17, 2024.
